# The effects of immediate programmed cryotherapy and continuous passive motion in patients after computer-assisted total knee arthroplasty: a prospective, randomized controlled trial

**DOI:** 10.1186/s13018-020-01924-y

**Published:** 2020-09-03

**Authors:** Mei-Chu Chen, Chiu-Chu Lin, Jih-Yang Ko, Feng-Chih Kuo

**Affiliations:** 1 Department of Nursing, Kaohsiung Chang Gung Memorial Hospital, Kaohsiung, Taiwan; 2 School of Nursing, Kaohsiung Medical University, Kaohsiung, Taiwan; 3 Department of Medical Research, Kaohsiung Medical University Hospital, Kaohsiung, Taiwan; 4 Department of Orthopedic Surgery, Kaohsiung Chang Gung Memorial Hospital, No. 123, Dapi Rd., Niaosong Dist., Kaohsiung, 833 Taiwan; 5 College of Medicine, Chang Gung University, Kaohsiung, Taiwan; 6 Center for Shockwave Medicine and Tissue Engineering, Kaohsiung Chang Gung Memorial Hospital, Kaohsiung, Taiwan

**Keywords:** Postoperative nursing intervention, Total knee arthroplasty, Continuous passive motion, Cryotherapy, Computer-assisted

## Abstract

**Background:**

The postoperative nursing intervention with immediate cryotherapy and continuous passive motion (CPM) remains elusive regarding the postoperative pain and range of motion (ROM) for patients undergoing computer-assisted total knee arthroplasty (CAS-TKA).

**Methods:**

A prospective, randomized controlled trial with a purposive sampling method was utilized. Sixty patients scheduled for a unilateral CAS-TKA at a medical center were randomly assigned to the intervention group (*n* = 30) and control group (*n* = 30). The intervention group applied programed cryotherapy and CPM within 1 h while returning to the ward on the day of surgery, while the control group did not. Data were analyzed using mixed models to compare the numeric rating scale (NRS) for pain, ROM, and swelling at postoperative day (POD) 4.

**Results:**

There was no significant difference in the NRS score between the groups (*p* = 0.168). The intervention group had significantly higher ROM than the control group (98° vs. 91°, *p* = 0.004) at POD 4. Although no significant difference in joint swelling was found between groups (*p* = 0.157), the intervention group had lower mean joint swelling (32.2 cm) than the control group (33.9 cm).

**Conclusions:**

Immediate programmed cryotherapy and continuous passive motion could help to improve ROM quickly after CAS-TKA. It should be incorporated into the daily nursing plan for patients undergoing CAS-TKA.

**Trial registration:**

ClinicalTrials.gov, NCT04136431. Registered 23 October 2019—retrospectively registered

## Background

Total knee arthroplasty (TKA) is an effective intervention in reducing pain and enhancing physical mobility for end-stage knee osteoarthritis and other synovial diseases [1–3]. Computer-assisted surgery (CAS) has shown to improve the restoration of the mechanical axis and gives better implant positioning [4]. In CAS-TKA, the proximal pins can be placed through the same incision, being more generous with the proximal part of the access. Distal or tibial pins are placed through separate stab incisions. Although the postoperative pain sensation after TKA can be controlled by oral/intravenous drugs combined with a peripheral nerve block (PNB) [5], local infiltration analgesia [6], or spinal anesthetic [7], the unexpected pain sensation and extremity swelling inhibit patients’ motivation for early mobilization, which results in prolonged hospital stays, delayed functional recovery, and negative psychological responses.

Pain has been defined as “an unpleasant sensory and emotional experience” associated with actual or potential tissue damage or described in terms of such damage [8]. Stiffness is affected by other factors after TKA, like early mobilization of the knee [9], implant position [10], rotational alignment [11, 12], size of the implant, overloading of some compartments [13], and previous range of motion (ROM) [14]. Therefore, adequate pain management and control of localized swelling and stiffness after TKA has become a priority because it is essential for improving patient satisfaction, prevention of complications, and enhancing quality of life by faster recovery. The current fast-track TKA apply proper pain management, avoid long PNB, and give physiotherapy instructions to start active motion as soon as possible [15]. Most of the current fast-track TKAs do not use continuous passive motion (CPM) because CPM implies a hospital longer stay. It is advisable to instruct patients to do rehabilitation at home with good pain management. Furthermore, the patient must be warned about the adverse effects of opioid drugs prescribed and should be taught to control these side effects to avoid the delay of the rehabilitation program [16].

CPM and cryotherapy can be useful for increasing early range of motion and to help to reduce swelling though no significant evidence of pain management can be shown after applying cryotherapy or CPM. The application of cryotherapy after TKA has been described extensively in the literature and it is standard care for inflammation disease, such as synovitis, arthritis, contusion, hematoma, dislocation, sprain, and swelling after surgery, to reduce swelling [17–19]. However, its benefits and value remain controversial due to the disparity in practice, such as differences in clinical protocols and the type of cryotherapy application [20]. CPM is a motorized device, which passively moves the knee joint within a certain degree to accelerate motion after knee fracture [21]. But the effects of CPM remain contentious in the literature [22] and rarely used now. Although controversial, cryotherapy and CPM have been used extensively as part of the standard postoperative management protocol for TKA patients without knowing its cost-effectiveness. However, the value of combined therapy of cryotherapy and CPM remains uncertain and unclear following CAS-TKA.

The hypothesis of this study was if the patients with immediate programmed cryotherapy and CPM had experience less postoperative pain, joint swelling, and increase ROM following CAS-TKA.

## Materials and methods

### Study design

A prospective, randomized, single-blinded controlled trial (ClinicalTrials.gov identifier, NCT04136431) was conducted at a single, academic, teaching, and medical hospital. After approval by the Institutional Review Board (IRB number 201102015B0), this study started to enroll the participants after obtaining written informed consent. All patients were enrolled by the Consolidated Standards of Reporting Trials.

### Participants and setting

Patients undergoing primary CAS-TKA were assessed for eligibility from January 2017 to July 2017. Patients between 18 and 90 years of age who underwent primary, unilateral navigation-assisted TKA were included in this study. The exclusion criteria were as follows: (1) patients who underwent bilateral TKAs, unicompartmental TKA, or revision TKA; (2) patients who had to remove previous implants or history of high-tibial or distal femoral corrective osteotomy; and (3) patients who were unable to response the questionnaires.

**Fig. 1:**
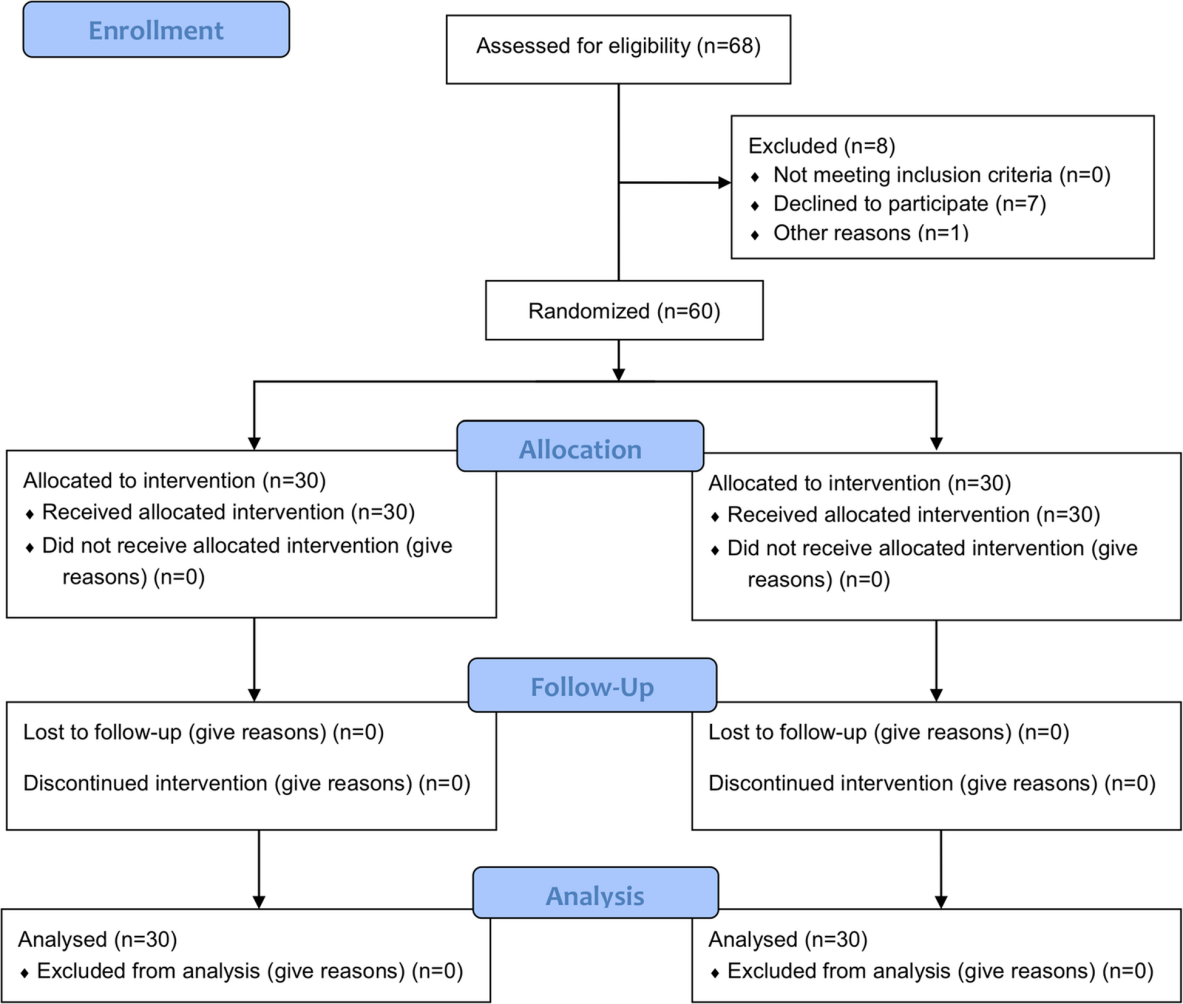
The CONSORT flowchart showing enrollment and exclusion of patients through the trial

A total of sixty-eight patients who underwent CAS-TKA were enrolled. Among them, seven patients declined to participate. One patient was excluded due to consciousness disturbance after returning the ward and unable to answer questions listed in the questionnaires. Therefore, 60 participants were randomly allocated to the intervention group (*n* = 30) and the control group (*n* = 30). All patients completed the analysis before discharge (Fig. 1).

### Randomization

The randomization was performed by the research staff using a parallel, 1 to 1 allocation method. A computer-generated randomization schedule was generated to assign participants to the intervention group by using a block size of 8 (1:1 ratio). The randomization occurred on the day of surgery using an opaque, sealed envelope, pre-labeled method.

### Surgical technique

All patients received a unilateral primary CAS-TKA by a single experienced surgeon. A pneumatic tourniquet was inflated to 300 mmHg pressure before the incision and deflated at the end of surgery after skin closure. No patient received preoperative tranexamic acid. The distal femur and proximal tibia bone cut were performed in an extramedullary manner. The navigation system was Vector Vision (BrainLab, Heimstetten, Germany), and all TKAs were cemented using the same prosthesis (NexGen Legacy posterior-stabilized prosthesis; Zimmer Inc., Warsaw, IN, USA). There was no local infiltration of the local anesthetic in the knee joint. A suction drain was inserted before wound closure and removed on postoperative day (POD) 1. All surgical wounds, including the stab incisions for the insertion of temporary pins, were closed with interrupted skin stitches. All patients received aspirin (100 mg once daily) as venous thromboembolism (VTE) prophylaxis for 14 days. Every patient was encouraged to do early and protected weight-bearing after the surgery on the same day or the POD 1 to avoid VTE complications [23]. Mechanical prophylaxis, such as intermittent pneumatic compression, was not performed in this study. Multimodal pain management, such as acetaminophen, cyclooxygenase-2 inhibitors drugs, and tramadol/acetaminophen combination tablets [Ultracet], was applied for all patients.

### Intervention group

In the intervention group, the systematic nursing intervention was implemented for the patients. The intervention group started to use a CPM machine and applied programed cryotherapy intermittently within 1 h while returning to the ward on the day of surgery. The CPM machine was set to move from 0° of extension to 60° of flexion. The application of programed cryotherapy was continued for 20 min and then stopped for 30 min. The programed cryotherapy with the cryotherapy pack was replaced every 4 h. From POD 1 to the day of discharge, the application of CPM and cryotherapy were conducted as the ward routine practice. The participants were blinded after assignment.

### Control group

For the control group, a routine nursing procedure was conducted, but the CPM device and cryotherapy were not applied on the day of surgery. The patients started CPM from 0° of extension to 60° of flexion and received cryotherapy on POD 1. However, the application of cryotherapy was not programed, which meant the frequency and intervals of cryotherapy were not specified and determined by the patients or caregivers.

### Data collection

The patients, outcome investigator, and statistician were blinded. Because of the different frequency of the replacement of the programed cryotherapy on POD 1, it was impossible to blind the surgeon and the floor nursing staff. The following participant characteristics (age, sex, body mass index, religion, and education), surgical variables (past surgical history, anesthesia method, wound length), the drainage amount, and the use of patient-controlled analgesia were collected.

### Primary and secondary outcomes

A numeric rating scale (NRS) with 0–10 points [24] was the primary outcome of interest in this study. It was evaluated when 0 is no pain and 10 the worst imaginable pain. The secondary outcome included the short-form McGill Pain Questionnaire (SF-MPQ), ROM, and the swelling status of the extremity. The SF-MPQ has been used to describe pain, feeling, memory, and influence in 15 pain situations [25]. The SF-MPQ has been validated to have a high correlation coefficient with long-form MPQ. A universal goniometer was used to measure ROM of the knee joints. The measurement of the thigh circumference was performed 15 cm proximal to the superior pole of the patella with a measurement tape, meanwhile 15 cm distal to the inferior pole of the patella for the calf circumference. Both thigh and calf circumference were compared to the normal contralateral leg to determine the amount of postoperative swelling. The above measurement was conducted at POD 1 and POD 4 or until the patients were discharged. All the patients completed the outcome evaluation by a research assistant.

### Statistical analysis

The sample size and Cohen’s effect size were calculated with the G*Power software package (version 3.1.4). The effect size *f* was interpreted as follows: *f* = 0.10 small effect, *f* = 0.25 medium effect, and *f* = 0.40 large effect. The collected data were coded with numbers and input to computer software. According to the research purpose and variable characteristics, IBM SPSS Statistics for Windows (version 22.0; IBM Corp., Armonk, NY, USA) was used for information analysis. The statistical methods included descriptive statistics and deductive statistics. Descriptive statistics involved (1) frequency and percentage, including the category and ordinary variables of basic features of the investigated subjects, such as gender, education background, religious belief, and past surgical history, and (2) mean value and standard deviation, including the equal-interval variables of basic features such as sex, education background, religious belief, operation experience, and anesthesia. Also, the scale score was described before and after measures. Deductive statistics included the independent *t* test and Chi-square test before measures, as well as the homogeneity test of basic features in the intervention group and control group. An independent *t* test was conducted to compare the pre-test difference between the two groups, and a paired *t* test of the samples was conducted to compare changes in the pre-test and post-intervention groups. ANCOVA (analysis of covariance) was used to compare pain after the treatment, as well as differences in the post-test scores of knee flexion and knee swelling. A *p* value < 0.05 was considered significantly different.

## Results

There was no significant difference in patients’ demographic data, past surgical history, anesthesia method, wound length, drainage amount, and the pain controller. Although there was not significantly different, the use of patient-controlled analgesia was reduced gradually in the intervention group (Table 1).

**Table 1:** Demographic data between the two groups

Demographics	All (*N* = 60)	Intervention group (*n* = 30)	Control group (*n* = 30)	*p* value
Age, year (SD)				.694
40–50 years	2 (3.3)	1 (3.3)	1 (3.3)	
51–60 years	11 (18.3)	7 (23.3)	4 (13.3)	
61–70 years	21 (35.0)	10 (33.3)	11 (36.7)	
71–80 years	25 (41.7)	11 (36.7)	14 (46.7)	
81–90 years	1 (1.7)	1 (3.3)	0 (0.0)	
Sex, *n* (%)				.519
Male	12 (20.0)	7 (23.3)	5 (16.7)	
Female	48 (80.0)	23 (76.7)	25 (83.3)	
Religion, *n* (%)				.511
No religion	7 (11.7)	5 (16.7)	2 (6.7)	
Taoism	24 (40.0)	13 (43.3)	11 (36.7)	
Buddhism	24 (40.0)	9 (30.0)	15 (50.0)	
Christian	3 (5.0)	2 (6.7)	1 (3.3)	
Others	2 (3.3)	1 (3.3)	1 (3.3)	
Education, *n* (%)				.911
Un literate	25 (41.7)	13 (43.3)	12 (40.0)	
Elementary school	20 (33.3)	10 (33.3)	10 (33.3)	
Middle school	7 (11.7)	3 (10.0)	4 (13.3)	
High school	4 (6.7)	2 (6.7)	2 (6.7)	
Specialist	3 (5.0)	1 (3.3)	2 (6.7)	
Above university	1 (1.7)	1 (3.3)	0 (0.0)	
Past surgical history, *n* (%)	2 (3.3)	1 (3.3)	1 (3.3)	
No	15 (25.0)	7 (23.3)	8 (26.7)	
Yes	44 (73.7)	23 (76.7)	21 (70.0)	
Missing value	1 (1.7)	0 (0.0)	1 (3.3)	
Anesthesia, *n* (%)				.301
General	56 (93.3)	27 (90.0)	29 (96.7)	
Regional	4 (6.7)	3 (10.0)	1 (3.3)	
Wound length, cm (SD)	14.2 (1.6)	13.9 (1.2)	14.5 (1.8)	.136
Drainage amount, ml (SD)	116 (59)	122 (66)	109 (52)	.387
Patient-controlled analgesia, n (%)	8 (13.3)	3 (10.0)	5 (16.7)	.448
The time of analgesics injection				
POD 1	1.0 (0.7)	0.9 (0.6)	1.2 (0.8)	.098
POD 2	0.4 (0.6)	0.3 (0.5)	0.5 (0.6)	.064
POD 3	0.1 (0.3)	0.03 (0.2)	0.2 (0.4)	.090

*POD* postoperative day, *SD* standard deviation

There was no significant difference in the NRS score between groups (*p* = 0.168). As shown in Table 2, SF-MPQ showed no significant difference in 15 pain situations of the tested patients between the two groups (*p* > 0.05). The average pain intensity of all the tested patients was lower than 2 points, indicating there was no serious pain (Table 2).

**Table 2:** The short-form McGill pain questionnaire between the intervention group and control group

Pain description	All (*N* = 60)		Intervention group (*n* = 30)		Control group (*n* = 30)		*p* value
	Mean	SD	Mean	SD	Mean	SD	
Throbbing	1.00	0.94	0.90	0.96	1.10	0.92	.414
Sudden sharp	0.45	0.83	0.57	1.04	0.33	0.55	.283
Stabbing	0.43	0.72	0.57	0.82	0.30	0.60	.155
Lancinating	0.27	0.66	0.20	0.55	0.33	0.76	.439
Spastic	0.20	0.55	0.20	0.55	0.20	0.55	1.000
Gnawing	0.22	0.56	0.17	0.53	0.27	0.58	.490
Burning	0.10	0.40	0.07	0.37	0.13	0.43	.522
Soreness	0.93	0.99	0.73	0.94	1.13	1.01	.118
Heavy feeling	1.33	1.05	1.27	1.08	1.40	1.04	.628
Pain upon touching	0.10	0.40	0.07	0.37	0.13	0.43	.522
Tearing	0.13	0.47	0.07	0.37	0.20	0.55	.274
Exhausted	0.87	1.03	0.67	1.03	1.07	1.01	.135
Unbearable	0.60	0.85	0.57	0.94	0.63	0.76	.764
Fear	0.37	0.71	0.50	0.82	0.23	0.57	.149
Tortured	0.32	0.65	0.37	0.76	0.27	0.52	.556

*SD* standard deviation

After adjusting patients’ demographic and surgical variables, the average ROM of the intervention group was significantly higher than the control group (98° vs. 91°, *p* = 0.007) at POD 4 (Table 3). Although no significant difference in joint swelling was found between groups (*p* = 0.157), the intervention group had lower mean joint swelling (32.2 cm) than the control group (33.9 cm).

**Table 3:** Comparison the degrees of range of motion after interventions in the pre-test (postoperative day 1) and post-test (postoperative day 4)

Group	Pre-test		Post-test (before correction)		Post-test (after correction*)	
	Mean	SD	Mean	SD	Mean	SE
Intervention	65	14.1	99	8.0	98	1.4
Control	56	11.6	91	7.8	91	1.4

*SD* standard deviation, *SE* standard error

*Patient’s demographic and surgical variables were corrected in this analysis

## Discussion

In past studies, CPM devices and cryotherapy have been applied to patients undergoing TKA with different intervention effects. However, no literature has discussed the effect of combined immediate programmed cryotherapy with CPM in patients following CAS-TKA. The findings showed that the intervention effect on ROM had significant differences, while the effect on pain and knee joint swelling had no significant difference.

The study indicated that pain intensity is a concern in TKA patients [26]. In this paper, the research results showed that constructive systematic nursing measures have significantly different effects on pain intensity after TKA. The literature indicated that patients who undergo computer-navigated surgery do not suffer from a serious loss of blood or pain because intramedullary nailing of the femur is not required for CAS-TKA [27]. The findings showed that the use of CPM devices can relieve pain intensity after surgery and that it is an appropriate rehabilitation exercise for alleviating pain. This finding was similar to the studies by Pozzi et al. [28]. Of the 15 types of pain, the respondents in the two groups mainly experienced throbbing pain, and a heavy feeling after CAS-TKA and the effective evaluation was dominated by feeling exhausted. The mean value of these three items had the highest score, ranging from 1 to 2. Given the results after CAS-TKA, health education should be enhanced to feel the nature of pain and teach the patients how to alleviate their pain and solve their physiologic discomfort. Interventions should be implemented for the three items to solve clinical problems.

CPM effect focuses on immediate use after surgery and not the amount of time needed for CPM [29]. In this study, the CPM intervention time was the operation day, and the length of use did not empathize. CPM can relieve pain intensity if it is instituted as early as possible. This finding was the same as the results in the literature. Pain intensity was alleviated on the fourth day after the operation. A recent meta-analysis suggested the use of CPM did not have a statistically significant improvement on ROM nor the functional outcomes after TKA [30]. In Taiwan, CPM is a standard therapy after TKA. A study from Taiwan also demonstrated that early application of CPM with initial high flexion angle and rapid progress significantly improve the ROM and functional outcome at the 3-month and 6-month follow-up [29]. But these studies evaluated the CPM effect on conventional TKA. Magin reported 100 consecutive CAS-TKA with early use of CPM and found a mean ROM of 110° on the day of discharge and 125° at 3 months after surgery [31]. Alkire and Swank was the only study to compare the benefits of CPM for patients undergoing CAS-TKA [32]. They found ROM was not different for those with CPM and those without CPM use at 2 weeks, 6 weeks, and 3 months postoperatively. The result of the immediate use of CPM in our study was contrary to Alkire and Swank [32]. We used immediate cryotherapy for pain relief, which was not described or used in Alkire’s study. However, we only evaluated inpatient outcomes nor short (3-month) or midterm (6-month) outcomes. It is necessary to establish one set of standard cryotherapy processes, ensure a uniform guidance mode, and monitor the effect of CPM intervention time. Many studies have separately discussed the effect of CPM and cryotherapy, but no studies have combined the two. In clinical manifestation and practice, the combination should be addressed after the revision of the research design. The effect of constructive systematic nursing interventions on ROM reached a significant difference. Early rehabilitation effect has demonstrated a better knee joint motion and performance [33]. These results were the same as the effect on ROM in this study. This study focused on early interventions of CPM and the resultant effect was different when CPM was used on the same day after CAS-TKA.

To sum up, constructive systematic nursing interventions have a significant effect on ROM. The result of the productive systematic nursing intervention on swelling showed no significant differences. Literature has demonstrated that the use of cryotherapy has a better effect on the wound swelling in TKA patients [34]. The results of pre-test swelling and post-test swelling of the two groups showed that the knee joint swelling of the intervention group was smaller on the first day and the fourth day than the control group, with a swelling difference of 1.82 cm. Theoretically, cryotherapy can reduce the excitability of free nerve ending and peripheral nerves, thereby indirectly reducing tissue edema and increasing the pain threshold to reduce pain intensity [34, 35].

### Limitations

In the previous study, due to limitations of the research framework and number of samples, the intervention effect on pain intensity and ROM had significant differences, while the impact on knee joint swelling had no significant difference. The study, measures, and subjects of this study knew which patients were distributed to the intervention group and the control group. In addition, this was not the first time of some participants undergoing TKA, and they knew that CPM would be used on the first day after operation instead of the same day of surgery. Thus, the subjects may have been prone to the Hawthorne effect. If a participant was randomly distributed to the intervention group, he/she must be informed to receive instruction. External validity may, therefore, have been affected. The study was conducted in a hospital center in southern Taiwan. The characteristics of the patients may be different from other places. Furthermore, the analysis found that the measurement tools were not sensitive, and the measurement scope for joint swelling was not clear. It is suggested that PQRST (provocation/quality/radiates or refers/severity/time) evaluation can be used in clinical practice to evaluate the pain of TKA patients, which can be considered during the evaluation of the intervention. Finally, we do not collect preoperative pain scores and duration into consideration, which might influence the outcome. If the patient had experienced pain in the preoperative period, the patient could somatize central pain and not benefit from the use of CPM and cryotherapy. The strength of this study was that it was a prospective and randomized controlled trial from a single surgeon with a standard protocol, which may eliminate other surgical factors related to the outcomes.

## Conclusion

Immediate programmed cryotherapy and continuous passive motion could help to improve ROM quickly after CAS-TKA. The establishment of this application should be incorporated into the daily nursing plan.

## Data Availability

Not applicable.

## Abbreviations

TKA: Total knee arthroplasty
CAS: Computer-assisted surgery
CPM: Continuous passive motion
ROM: Range of motion
POD: Postoperative day
NRS: Numeric rating scale
SF-MPQ: Short-form McGill Pain Questionnaire
